# Language testing in awake craniotomy for brain tumor resection: A survey of current perioperative practice in the United Kingdom

**DOI:** 10.1093/nop/npaf027

**Published:** 2025-03-08

**Authors:** Sonia Mariotti, Neil U Barua, T R Williamson, Hajira Mumtaz, Kris Kinsey, Anna E Piasecki

**Affiliations:** Neurosurgery Department, Southmead Hospital, North Bristol NHS Trust, Bristol, UK; Brain, Language and Behaviour Laboratory, University of the West of England, Bristol, UK; Neurosurgery Department, Southmead Hospital, North Bristol NHS Trust, Bristol, UK; Brain, Language and Behaviour Laboratory, University of the West of England, Bristol, UK; Neurosurgery Department, Southmead Hospital, North Bristol NHS Trust, Bristol, UK; Brain, Language and Behaviour Laboratory, University of the West of England, Bristol, UK; Neurosurgery Department, Southmead Hospital, North Bristol NHS Trust, Bristol, UK; Brain, Language and Behaviour Laboratory, University of the West of England, Bristol, UK; Brain, Language and Behaviour Laboratory, University of the West of England, Bristol, UK; Neurosurgery Department, Southmead Hospital, North Bristol NHS Trust, Bristol, UK; Brain, Language and Behaviour Laboratory, University of the West of England, Bristol, UK

## Abstract

**Background:**

Language testing and mapping procedures are considered the gold standard for safe tumor resection and preservation of language and communication in patients with tumors located in an area eloquent for language, especially in the presence of low-grade gliomas. However, the current status of language testing in awake craniotomy in the United Kingdom is unknown. The main aim of this study was to describe the language testing practices in awake brain surgery across the United Kingdom.

**Methods:**

An online survey was addressed to medical practitioners working with brain tumor patients during the phases of language testing. Questions inquired about the tests and approaches for language testing before, during, and after the surgery. The survey also explored the management of bilingual (for the sake of simplicity, the term bilingual is used throughout the article to refer to patients who speak 2 or more languages) brain tumor patients and gathered personal perspectives from clinicians.

**Results:**

Responses were obtained from 37 clinicians. Speech and language therapists and neuropsychologists administered language tests to patients, and those with sufficient language skills for completing intraoperative tests were eligible for awake mapping. A combination of standardized language batteries and homemade tasks were used for language testing, leading to variability in testing practices across institutions. For language mapping, the most popular tasks were picture naming, sentence completion, and repetition. Object and action naming were used across both the monolingual and bilingual patient groups. The timing of postoperative assessments varied according to patient needs and clinician availability. Bilingual patients were evaluated with interpreters and limited materials, compared to monolinguals.

**Conclusions:**

The provision of awake craniotomy language testing presents differences across UK-based institutions. Responders advocate for more comprehensive, updated, and inclusive materials to facilitate language testing in modern patient cohorts spanning a wide range of linguistic skills and foreign languages.

**Key Points**

- Language testing practices in awake craniotomy vary across the United Kingdom.
- National guidelines and protocols should be implemented to provide equal care.
- Updated and comprehensive tasks are needed to test patients with diverse linguistic skills and languages.

**Importance of the Study**

The article provides an unprecedented overview of neurosurgical language testing practices in the United Kingdom. While brain cancer patients in the United Kingdom receive varying treatments depending on the center, there is little information on perioperative language testing protocols. This study addresses that gap by summarizing the results of 37 surveys from clinicians working with brain tumor patients in England, Scotland, Wales, and Northern Ireland. It highlights common trends, such as the role of clinicians in administering language tests and the scarcity of resources, as well as differences in testing practices across centers. The lack of standardized guidelines leads clinicians to create their own tests, particularly for patients who do not meet typical criteria for awake brain surgery, such as those with reduced communicative abilities. Challenges in testing bilingual patients are also noted. The study emphasizes the need for standardization and national guidelines to ensure equal patient care across the United Kingdom.

In the United Kingdom, 34 people are diagnosed with brain cancer every day^1^ and this number is even higher if including people with secondary brain tumors or non-cancerous brain tumors, for which reliable data is lacking. Without intervention, brain tumors in language-eloquent areas increase the risk of developing language and cognitive impairments, such as word-retrieval difficulties, impaired comprehension,^2^ semantic dysfluency,^3^ and a generalized language decline.^4^ To minimize these risks, patients are offered awake brain surgery (or craniotomy) for the removal of tumor tissue. During surgery, the patient is woken from anesthesia and language testing is performed by specialist speech and language therapists or neuropsychologists. Direct electrical stimulation (DES) of the eloquent cortical and subcortical structures results in temporary language errors allowing the extent of tumor removal to be defined by functional boundaries.^5^ This technique is known as language mapping and helps to identify and preserve language-functional brain areas.

The fundamental aim of awake craniotomy is maximal safe tumor resection, as the extent of resection is consistently associated with prolonged survival and reduction of cancer recurrence rate, particularly in gliomas, either low-grade or high-grade.^6^ However, one of the major challenges of intrinsic brain tumor surgery is differentiating brain tumors from adjacent functional brain tissue. Resection beyond the brain/tumor interface can result in irreversible neurological deficits which adversely affect quality of life and survival.^7^ It is therefore imperative that the oncological aims of surgery are balanced with the preservation of neurological function, and this is particularly important for patients with brain tumors in language-eloquent areas where the postoperative loss of communication can result in social isolation, reduced independence, disqualification from chemo-radiotherapy and adversely impact the quality of life.^8–10^

Although language mapping in awake craniotomy has been practiced for decades, recent surveys and reviews^11–14^ highlight significant variability across European and international institutions. Differences in practice across countries are expected due to varying regulatory bodies and scarcity of underpinning research, but less variation in patient care at a national level is desirable to ensure consistent treatment and outcomes. At present, the status of language testing practices in brain tumor surgery across the United Kingdom is unknown. This work, to the best of our knowledge the first of its kind, reports the results of a nationwide survey, gathering insights into how language testing in awake craniotomy is performed under the auspices of one common national health system.

## Methods

The survey questions (available at OSF) were developed based on evidence of reviews^15,16^, case reports,^17,18^ and previous surveys in awake craniotomy (AC).^12,19^The survey’s structure mirrored the brain tumor patient pathway for language assessments in awake surgery, covering preoperative (7 questions), intraoperative (19 questions), and postoperative (5 questions) phases. The survey questions were designed to elicit eligibility criteria, sociodemographic information, and details about their institutions and patients (11 questions). Two open-ended questions for personal reflections were also included. Questions (45 in total) were mostly multiple-choice with some open-ended ones. Prior to survey distribution, the design and questions were piloted with a neurosurgeon and a speech and language therapist involved in awake craniotomies. The involvement in the pilot phase excluded their participation in the main study.

The survey was approved by the University of the West of England’s Research Ethics Committee (Ref no: CATE-2324-206) and distributed in November 2023 to all neurosurgical units in the United Kingdom.^20^ Recipients were healthcare professionals involved in awake craniotomy language testing across the United Kingdom, including, but not limited to, neurosurgeons, neuropsychologists, and speech and language therapists. The survey was shared via personal connections, social media, and networks, such as the Tessa Jowell Brain Cancer Mission and the British Neuro-Oncology Society. Multiple responders from the same institution could participate, reflecting various professional disciplines.

Complete surveys were considered for the final analysis, as it was not possible to compare questions where clinicians only provided partial responses. Responses were analyzed using descriptive statistics. For single-choice questions, we report percentages and frequencies, depending on the measure that better describes the answers. In multiple-choice questions, the percentages indicate the proportion of respondents who selected each option, meaning that the total of responses across all options does not add up to 100%. Free-text boxes were provided after each question, to capture additional relevant comments or other suggestions provided by responders.

## Results

### Responder Characteristics

Responses were obtained from 37 clinicians working in AC language testing across the United Kingdom. The option to indicate the institution was not mandatory, but was indicated by 20 clinicians who worked in England (16), Scotland (2), Wales (1), and Northern Ireland (1) (Supplementary Table 1). Table 1 shows the responders’ specialty and years of experience, their institutions, and the frequency of ACs per year.

**Table 1.** Responders and Their Institutions (37)

Profession	
Speech and language therapist	19 (51.3%)
Neurosurgeon	10 (27.1 %)
Neuropsychologist	7 (18.9 %)
Neuroscientist	1 (2.7 %)
*Years of experience*	
<5 years	10 (27.1 %)
5–10 years	7 (18.9 %)
10–20 years	12 (32.4 %)
>20 years	8 (21.6 %)
*Institutions*	
University hospitals	31 (83.8%)
Community hospitals*	6 (16.2%)
*With occasional private practice cases	
*Annual awake craniotomies*	
<8	8 (21.6 %)
10–20	20 (54.2 %)
20–30	4 (10.8 %)
>30	4 (10.8 %)
No answer	1 (2.7 %)

Responders’ details (profession and years of experience) and information on the type of institutions they work at and the frequency of surgeries performed annually in their centers.

Responders indicated that language tests in their institutions are administered by speech and language therapists (SLT, 75.6%), neuropsychologists (51.3%), and other roles (13.5%) such as physicians assistants, neuroscientists, neurosurgeons, and neurosurgical trainees. In 75% of cases, language tests are administered by the same clinicians at pre-, intra-, and postoperative phases.

### Eligibility for Awake Surgery

The majority of responders (97.2 %) agreed that patients needed to have at least sentence-length communication to be eligible for awake surgery. All responders agreed that patients communicating non-verbally are ineligible. Comments specified that when patients cannot complete preoperative assessments, materials are adjusted to match their language abilities for intraoperative testing.

Awake craniotomy is performed when brain tumors are located in or in proximity to language-responsible areas of the brain, mostly in the left hemisphere. For tumors located in the right hemisphere, responders reported that AC is offered if patients are left-handed (10.8%), with evidence of right-/bi-hemispheric language dominance, with, for example, confirmation with fMRI, (40.5%), or for any patient (27.1 %). A portion of clinicians (18.9 %) would map the right hemisphere when sensation or motor functions are considered at risk, with language being tested if baseline assessments indicate crossed lateralization.

### Preoperative Assessment

The testing material for preoperative language assessments comprises standardized language batteries and homemade experimental tasks. Clinicians use standardized language batteries (37.2 %), such as the Comprehensive Aphasia Test^21^ (CAT) and Boston Naming Test^22^ (BNT), high-level language tests, such as the Mount Wilga^23^ and Brisbane Evidence-Based Language Test^24^ (12.8 %), and tasks developed in their institutions (19.2 %). Other tests (15.4 %) include the Sheffield Language Screen,^25^ tasks from the Dutch Linguistic Intraoperative Protocol^26^ (DuLIP), and the verb and noun test (VAN-POP).^27^ Clinicians also use standardized cognitive tests for baseline evaluations, such as the Cognitive Linguistic Quick Test^28^ and the Montreal Cognitive Assessment^29^ (15.4 %).

#### Criteria for intraoperative test selection.—

The selection of intraoperative tests for language mapping and monitoring is not differentiated. Test selection was based on a number of criteria: tumor characteristics, patient language and cognitive abilities, patient characteristics, and occasionally, patient requests (Supplementary Table 2). The selection of intraoperative tasks with reference to the tumor location is based on knowledge of brain-language anatomo-functional correlations from the DuLIP and other models of language processing (63.6 %), from the neurosurgeon (54.4 %) and the multidisciplinary team’s experience (45.5 %). Other methods (18.2 %) include evidence from neuroimaging such as functional MRI and diffusion tensor imaging (DTI), and leveraging on neuropsychologists and speech and language therapists’ expertise.

### Intraoperative Tests

For intraoperative language assessments, responders develop bespoke tasks within their institution (72.9 %) or adopt homemade tasks shared by another institution/SLT service (10.8 %). Subtests from standardized batteries are used (54 %), such as the Oral image naming test (DO80^30^) and Pyramid and Palm Trees Test (PPTT^31^), alongside tasks translated from DuLIP (51.3 %).

During intraoperative language testing, 91.9% of responders perform subcortical mapping for tumors located in proximity to white matter tracts. However, 64.8% of clinicians do not differentiate language tasks for mapping and monitoring. Language monitoring tasks include repetition (24.3%), picture naming (14%), sentence completion (17%), counting (10.8%), and spontaneous/connected speech (35.1%). Tasks for mapping (only) linguistic functions in monolingual and bilingual patients are reported in Table 2. The most commonly used test was object naming (78.4%) for semantics, phonology, and bilingual language processing (75.7%). Action naming was also used to assess semantics (45.9%), syntax (56.7%), and bilingual language processing (45.9%).

**Table 2.** Testing Linguistic Functions Intraoperatively (37 Responders, Multiple Choice Option)

Task	Linguistic levels			*Bilingual language processing*
	*Semantics*	*Phonology*	*Syntax*	
*Object naming*	78.4%	✓		75.7%
*Action naming*	45.9%		56.7%	45.9%
Semantic association	67.6%			
*Sentence completion*	54.1%		51.3%	37.8%
Odd picture out	45.9%			
Logic/reasoning	16.2%			
*Word/non-word repetition*		64.8%		51.3%
*Odd word out*	✓	51.3%		
Rhyme judgement		35.1%		
Verb generation			37.8%	
*Grammaticality judgement*	✓		37.8%	
*Picture definition*	✓		✓	
*Conversation*			✓	✓
Other tasks:	word and picture definition	Sentence/pseudoword repetition, auditory naming	response to questions	reading, “all the same tests as for English”

Tasks for intraoperative testing of semantics, phonology, syntax and bilingual language processing. ✓ means responders mentioned this task in free text responses but no numeric data was available.

Italicized tasks are used to assess more than two linguistic functions.

Responders explained that writing, either by asking the patient to write or type, is assessed only in selected cases, during language mapping only (2.7 %), and both mapping and monitoring (10.8 %). Reading is tested more frequently than writing (67.6%), by sentence completion, word-to-picture matching, and reading aloud sentences, words, and nonwords. A clinician pointed out that many tasks contain a reading component in the stimuli and reading is therefore automatically assessed.

Other than spoken and written language, some responders (40.5 %) test aspects of non-verbal communication intraoperatively. These are facial expressions (24.3 %), head nodding (8.1 %), and gesturing (5.4 %). One responder reported testing sign language (2.7 %). Other responders (18.9 %) informally monitored facial movements, sensation, finger tapping, and administered a screwdriver task.

Other than language abilities, the responders’ institution offers mapping of other skills. These include motor planning (72.9%), visual perception (35.1%), arithmetic (29.7%), executive functions (27%), music (16.2%), memory (8.1%), and patient-specific skills such as drumming and sketching (27%).

Patients’ intraoperative performance was recorded by 46% of responders. This concerned tracking error type (44.1%), accuracy (32.4%), and response times (8.8%). Methods include using paper or tablet-based score sheets and having a second therapist or nurse mark patient performance. Other responders (14.7%) explained that record keeping happens inconsistently and informally, through feedback to the neurosurgeon and in post-surgery annotations. A clinician highlighted the difficulty of tracking response times without slowing down the testing procedure.

### Postoperative Follow-up

Postoperative language assessments are carried out within 1 week from the surgery for most responders (83.8%). Responders explained that the timing of the first postoperative language assessment after the surgery varies according to patient-specific factors. One responder argued that tests should be administered at least 7 days after the operation, but evaluations are mostly made after 2 days due to patients being discharged early. Another responder explained that it is inappropriate to conduct language assessments on the first postoperative day. Instead, in their institution, the SLT meets the patient the day after the surgery, providing reassurance and counseling. “The clinician would also conduct an informal assessment and summarize how the patient is communicating to compare it with the preoperative baseline. Formal assessments are then conducted 2–3 days postop.”

Additional follow-ups are conducted by 86.5% of clinicians, the timing of which varies according to patient presentation and clinicians’ availability (Supplementary Tables 3 and 4). The language tests to evaluate patients’ skills were the same as the same preoperative (48.6 %) or intraoperative tasks (5.4 %), or reduced testing if patients’ conditions suggested so (8.1%). Standardized batteries and informal assessments were also administered (37.8 %). A neuropsychologist reported using the same tests as preoperative, while the SLT in the same team might use the same tests as intraoperative instead.

### Bilingual Patients

**Figure 1.**
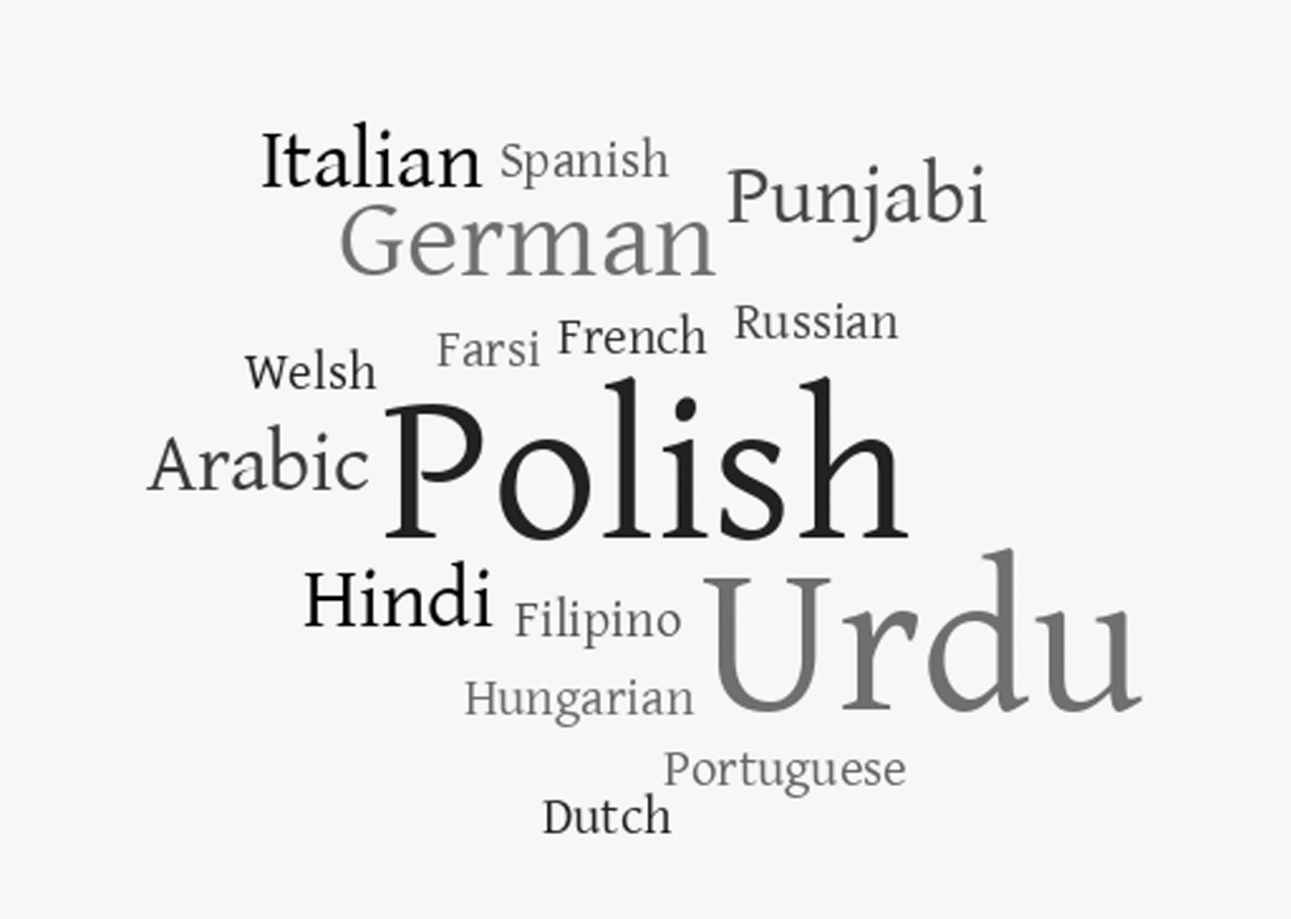
Foreign languages are spoken by bilingual patients. Bigger words correspond to more frequent languages.

Patients who speak more than one language were less than half, or the minority, of AC patients (81% of responders). Foreign languages spoken by bilingual patients vary and are represented in the word cloud (Figure 1; Supplementary Table 5).

At the preoperative evaluation, bilingual patients were tested both in their native language(s) and English by 59% of responders, while 14% reported English testing only. Other responses (27%) described testing patients in their different languages according to daily usage or only at the intraoperative stage. To assess bilingual language skills preoperatively, clinicians (32/37) used both objective, picture naming (75%), and subjective measures, patient (59.4%) and family’s opinion (40.6%). Free text responses highlighted the scarcity of tests for bilingual patients: clinicians used “Whatever is available in foreign languages,” specifying that “It depends on what is available in their [patients’] language. Usually, the options are extremely limited.” Interpreting services or bilingual clinical staff were also involved to facilitate testing.

For intraoperative bilingual testing, interpreters are routinely involved (67.6 %). Tests in languages other than English are used when available (27 %), and if not, tests in English are translated to the language(s) of interest (29.7 %). Nine responders reported testing in English only and 2 were unable to comment.

#### Language switching.—

Language switching (LS), bilingual patients’ ability to alternate between languages, is evaluated preoperatively by 46% of responders, using picture naming, Pyramids and Palm Trees Test, or informal observation in conversation with patients and family. A responder reported that “patients are asked to complete a set of tasks in English and then repeat the same tasks in the other language via interpreter […] however, language switching is not tested within one task.” Another clinician reported testing for switching “ad-hoc” but urged that it is “something that needs to be developed.” For the rest of responders who do not test language switching preoperatively (54%), some reported being “very aware that this happens, and is especially apparent intraoperatively as opposed to preoperatively.” Intraoperatively, 18.2% of responders test for LS. Postoperative pathological LS is reported by 16.2% of responders.

### Clinicians’ Perspectives

According to 75% of responders, there is a need for consensus in awake craniotomy language testing. Responders identified 5 key areas for improvement. First, guidance on brain region to language function mapping would be useful to aid intraoperative test selection based on tumor location (38%). Second, a standardized intraoperative protocol with diverse tasks (32%) would facilitate the assessment of all levels of language processing. Clinicians advocate for this protocol to be adaptable to new research and easily shareable among clinicians and researchers, plus allowing flexibility to accommodate individual patient needs. Third, updated tasks for high-level language functions are needed (23%) to test beyond simple picture naming and better suit modern patient cohorts. Fourth, some clinicians argued for more evidence for the effectiveness of diverse tasks (7%) to ensure that the language measures used effectively improve functional and survival outcomes. Finally, there is a need for technological tools to capture intraoperative data efficiently.

## Discussion

A nationwide survey was launched to reveal the current status of language testing in awake brain surgery in the United Kingdom. Responders (37) worked primarily in NHS institutions, teaching hospitals (83.8%), and community hospitals (16.2%). There are 32 neurosurgery centers in the United Kingdom^20^ and 16 were covered by the responders who provided information about their place of work (20). With 17 anonymous responses that could originate in any other center in the United Kingdom, the survey provides coverage of clinical practice across the country. In the United Kingdom, 34 people are diagnosed with brain cancer every day (Cancer Research UK, 2023), with awake brain surgery for tumor removal carried out in large numbers. More than half of centers perform between 10 and 20 surgeries per year (54.2 %), 8 centers perform less than 8 (21.6 %), and 8 centers perform more than 20 per year (21.6 %). These figures show that there is a variation in the number of cases performed in each center, ranging from more than 20 to less than 8. Currently, there is no consensus on the minimum number of procedures required to maintain skills and competence in awake craniotomy in the United Kingdom. Therefore, the potential impact of low case volume and the definition of minimum requirements is an area for future research.

In the United Kingdom, speech and language therapists (75.6%) and neuropsychologists (51.3%) are the healthcare professionals who administer language tests for the majority. Staff availability and service provisions for AC determine clinicians’ involvement, with physician’s assistants and neurosurgical trainees occasionally stepping in to administer language tests. Clinicians administering tests should be “language specialists” with knowledge of neurolinguistics and neuroanatomy of language processing,^32^ trained in scoring and interpreting test results. Inconsistent involvement of clinicians may affect the accuracy of assessments, potentially impacting surgical decisions and patient outcomes. Continuity in language assessments, both in terms of personnel and methods, is crucial^14^ and guaranteed in 75% of institutions. This is a positive outcome of the survey, suggesting that the majority of patients receive language tests administered by the same clinician.

According to the responders, patients with intact language skills are ideal candidates for awake craniotomy. On the contrary, patients with severe communicative impairments rarely undergo awake surgery, and non-verbal patients are typically ineligible. However, clinicians devise personalized tasks to be able to offer AC to patients who do not present with neurotypical communication. Cases of sign language users undergoing awake craniotomy are rare,^33–36^ with one recently reported in the United Kingdom, where British Sign Language was included in intraoperative mapping tasks.^37^ Urgently needed are guidelines and paradigms for patients with limited communication abilities, such as those communicating in single-word utterances, or non-verbally with gestures. Even in verbal patients, gestures and facial expressions are somewhat monitored informally by clinicians (40.5%), but this should be routinely done as they are integral aspects of language and communication.^38^

Concerning language tests for brain tumor patients, clinicians often use a “mix and match” approach. Similarly to what was reported in reviews,^14^ subsets from standardized language batteries, such as PPTT, BNT, and CAT, are selected along with bespoke measures, for both pre- and intraoperative phases. Additionally, to facilitate mapping in patients with impaired communication or to allow mapping of foreign languages in bilingual patients, existing tasks are modified accordingly by clinical teams. Two takeaways can be extracted from this: there is a limited availability of language tests for brain tumor patients (both monolingual and bilingual), and the existing ones are not always appropriate for awake craniotomy settings. For example, language batteries originally developed for other patient populations, such as brain injury and stroke patients, are used in the context of awake craniotomy,^9^ despite their inadequacy to capture subtle language changes characterizing brain tumor patients.^4^ Moreover, intraoperative tests require specific criteria to be suitable for mapping, such as direct electrical stimulation (DES) time constraint, ease of use, and the capacity to assess critical language functions,^39^ therefore, requiring adjustments to existing tests that do not meet these criteria. Designing tests that fulfill these criteria is time-consuming and resource-heavy, as it requires a methodical approach^40^ that incorporates a comprehensive understanding of linguistics and test development requirements. This may explain why there is a limited number of available tests and why creating novel or updated tasks often presents a challenge.

The most common intraoperative tasks according to the survey are object and action naming, used to assess semantics, phonology, syntax, and bilingual language processing. Picture naming paradigms have been widely validated in awake brain surgery for language mapping, as they effectively elicit language production and prevent anomia.^41^ However, this paradigm is often overused to test many linguistic functions,^12^ where more specialized tasks might offer more accurate assessments.^42^ Tailored tasks to language mapping (with DES) and monitoring (without DES) should also be adopted to address the different cognitive demands and task requirements in these 2 phases of intraoperative testing.^14^ Yet, more than half of responders reported using the same tasks, suggesting a need for more specialized materials for each phase.

For bilingual language testing, responders translate available tests to the language(s) of interest, involve interpreters, or resort to testing patients in English only. None of these options are desirable. Firstly, it is highly recommended to map all the languages spoken by the patient, as both shared and distinct cortical areas underpin bilingual language processing.^43^ Secondly, intraoperative testing should prioritize addressing language-specific features, rather than translating English-based criteria to other languages.^13,44^ However, despite the presence of bilingual intraoperative tasks, these are mostly limited to picture naming,^27,40,45,46^ therefore failing to assess a wider spectrum of language skills, including the specific bilingual ability of language switching. In the survey, language switching is evaluated preoperatively by approximately half of responders, but is inconsistently tested intraoperatively, despite clinicians noticing language switching difficulties during the surgery. Moreover, 17% of clinicians reported postoperative pathological switching. As a responder specified, tests for language switching need to be developed, as another clinician pointed out that asking patients to alternate between performing one task in English and the next task in the patient’s other language does not count as language switching. The decision to test for language switching remains contentious in the literature. Some neurosurgeons^47,48^ advocate for serial testing of languages, while other scholars^13^ argue for including language switching in intraoperative assessments, supported by specific tasks developed for this purpose.^49–51^ This ongoing debate underscores the urgent need for guidelines and tasks to ensure the effective preservation of bilingual skills in awake craniotomy patients.

Regarding the timing of postoperative language evaluations, the first assessment is conducted within a week of the surgery by more than 80% of clinicians, with formal assessments taking place within 3 days postoperatively. There seems to be a lack of agreement in the responses to our survey as to the best timing for postoperative testing. Some clinicians commented on the inappropriateness of testing patients so early after the surgery and others indicated that due to patient-related reasons, testing takes place on the second or third day after the operation. Despite the paucity of investigations on postoperative assessments, our results are in line with those emerging from European practice,^52^ where the majority of centers reported postoperative speech and language assessments at bedside (within 10 days from surgery). Further research and clear guidance may be needed to reach a consensus toward the best standard of care for patients.

Clinicians in the survey advocated for greater openness in sharing tests and approaches across institutions and among researchers and clinical staff. To achieve such collaboration, a shared digital platform for open exchange and resource sharing would facilitate communication between clinicians and institutions. Such a platform could lead to less variability in practice, ultimately benefiting both patients and practitioners. The call for collaboration aligns with findings in international practice,^12^ where consensus among disciplines and institutions was deemed crucial to enhance the validity and accuracy of intraoperative language mapping. To promote consistency in care and provide robust materials for language evaluation in awake brain surgery, a critical review of perioperative tests is needed. For instance, clinicians require updated tasks, beyond single-word picture-naming exercises and moving away from “outdated, no longer culturally relevant” tests such as the Boston Naming Test (developed in 1983),^22^ as a responder commented, to better suit modern patient cohorts.

Developing a standardized but flexible protocol for testing language skills would fill the current gap in AC language testing in the United Kingdom. A common protocol would provide clear guidance and tasks for clinicians to use when planning and administering tests, while also incorporating the latest research on the neuroanatomy of monolingual and bilingual language processing. A shared national approach would ensure healthcare equality, consistent patient experiences, and comparable surgical outcomes, while also enhancing research efforts by creating a more uniform framework for collecting and analyzing data across different institutions.

## Limitations

This study’s findings are based on surveys completed by 37 clinicians, providing a snapshot of language testing practices in the United Kingdom. The number of responders might have been higher with a shorter survey and a longer time for completion. Similarly, the structured nature of the questions may have prevented responders from providing insights on other aspects of ACs, which could be the subject of future investigations. The survey’s reliance on reported data is a noted limitation, but the collected evidence still offers valuable insight into current language testing practices in the United Kingdom.

## Conclusions

The survey revealed quantitative and qualitative differences in the provision of awake craniotomy language testing across the United Kingdom. While some variability in awake brain surgery is inevitable due to factors such as patient diversity and clinician expertise, standardized testing materials and national guidelines could help promote more consistent and equitable care. Most clinicians in the survey are aware of the challenges and are working with available resources to improve the postoperative quality of life for brain tumor patients. Their efforts, however, require greater support in terms of materials and guidance from the medical and research community, and national policymakers.

We encourage researchers and clinical teams abroad to investigate the status quo of perioperative language testing within their respective countries. As with the present work, focusing on national clinical practices, rather than broader European or international surveys, may better reveal hidden disparities and challenges within healthcare systems, providing an opportunity to address specific patient needs in each country. International collaboration, on the other hand, could better serve bilingual patients, whose language testing is currently too diverse and limited. Clinicians from different countries should be open to coming together in developing and sharing language tests, to increase the availability of relevant testing materials in multiple languages.

## Supplementary material

Supplementary material is available online at *Neuro-Oncology Practice* (https://academic.oup.com/nop/).

npaf027_suppl_Supplementary_Tables_1-5

## Data Availability

All data generated from the survey are presented in the main manuscript text and the supplementary materials.
