# Supplementary material for: Language testing in awake craniotomy for brain tumor resection: A survey of current perioperative practice in the United Kingdom
Source: Neurooncol Pract. 2025 Mar 8;12(4):654–62. doi: 10.1093/nop/npaf027 (PMC12349761; doi:10.1093/nop/npaf027)
Supplement: npaf027_suppl_Supplementary_Tables_1-5 [file npaf027_suppl_supplementary_tables_1-5.docx]

## Supplementary materials

*Supplementary Table 1*

| **Responders’ institutions* (9)** |
| --- |
| NHS Lothian (Scotland) |
| University Hospital Southampton (England) |
| Ninewells Hospital, Dundee (Scotland) |
| University Hospitals Sussex NHS Foundation Trust (2x) (England) |
| University Hospitals of North Midlands (England) |
| National Hospital for Neurology & Neurosurgery(England) |
| Royal London Hospital (England)  Unspecified (Northern Ireland) |
| **Responders’ institutions awarded Tessa Jowell Centre for Excellence (11)** |
| The Walton Centre NHS Foundation Trust (England) |
| Leeds Teaching Hospitals Trust (2x) (England) |
| Southmead Hospital, Bristol (2x) (England) |
| Oxford University Hospitals (England) |
| St Georges University Hospital (2x) (England) |
| University College London Hospital (England) |
| King's College Hospital, London (England) |
| Velindre Cancer Centre (Wales) |

*Caption:* Location of responders’ institutions. The option to indicate the location was not mandatory. There are 32 adult neurosurgery centers in the UK, with 25 located in England (Society of British Neurosurgeons, 2024).

*Supplementary Table 2*

|  | **Selection of intraoperative tests**  (37 responders to the survey; multiple answers possible) | **Mapping** | **Monitoring** |
| --- | --- | --- | --- |
| Tumor characteristics: location, pathology, grade, size | | 33 (89.2 %) | 31 (83.7 %) |
| Patient profile: language abilities, cognitive status, stress level | | 29 (78.4 %) | 28 (75.6 %) |
| Patient characteristics: age, education, profession, bilingual status | | 26 (70.3 %) | 31 (83.7 %) |
| Patient's request | | 7 (18.9 %) | 8 (21.6 %) |
| Other: seizure/preoperative error pattern | | 6 (16.2 %) | 4 (10.8 %) |

*Caption:* Methods to select intraoperative tests for language mapping and monitoring.

*Supplementary Table 3*

| **First post-operative language assessment**  (37 responders) | |
| --- | --- |
| 1-3 days | 29 (78.4%) |
| 4-7 days | 2 (5.4%) |
| 1-2 weeks | 1 (2.7%) |
| Other: 6-12 months* | 5 (13.5%) |

*Clinicians might have misunderstood the question

*Caption:* Timing of first postoperative language assessment after awake craniotomy.

*Supplementary Table 4*

| **Additional follow-ups after the first one**  (32 responders; multiple responses possible) | |
| --- | --- |
| 1-3 days | 11 (34.4 %) |
| 4-7 days | 8 (25 %) |
| 1-2 weeks | 5 (15.6 %) |
| 2-4 weeks | 8 (25 %) |
| 1-2 months | 10 (31.2 %) |
| 6-12 months | 7 (21.9 %) |
| 1+ years | 2 (6.2 %) |
| Other/free text comments: | 11 (34.4 %) |
| *“It depends on postoperative function”*  *“Bespoke and according to patient need”*  *“Ideally 48 hours, 10 days and 3 months, but often this is impractical; patients who are not impaired at the first post-op assessment are not re-assessed at each time point”* | |

*Caption:* Timing of additional postoperative language assessment after awake craniotomy.

*Supplementary Table 5*

| **Foreign languages spoken by bilingual patients** | |
| --- | --- |
| **Languages** | **Occurrence** |
| Polish, Urdu | 5 |
| German | 3 |
| Italian, Hungarian, French | 2 |
| Welsh, Portuguese, Arabic, Spanish, Dutch, Farsi, Russian, Filipino, Gujarati | 1 |
| Eastern European* | 2 |
| Various European languages* | 1 |

*Caption:* Languages spoken by bilingual patients as reported by responders. *

“Eastern Eastern European” and “various European languages” were reported by responders but do not correspond to any specific languages.

*Supplementary Table 6*

|  |  | **Patients’ language skills for AC eligibility**  (multiple answers possible) | | | | |
| --- | --- | --- | --- | --- | --- | --- |
| Non-verbal/ communicating non-verbally | | | 0 | | (0%) |  |
| One-word utterances, gesturing | | | 2 | | (5.4 %) |  |
| Phrase-length utterances | | | 9 | | (24.3 %) |  |
| Sentence-length utterances | | | 21 | | (56.7 %) |  |
| Neurotypical-level communication | | | 15 | | (40.5 %) |  |
| Other/free text comments:  *“Patient must be capable of giving informed consent”*  *“Case by case” “It depends”*  *“Reliable baseline for intraoperative mapping”* | | | | 10 | (27.1 %) |  |

*Caption:* Languages abilities of eligible patients for awake craniotomy.

*Supplementary Figure 1*

**Patients’ age**


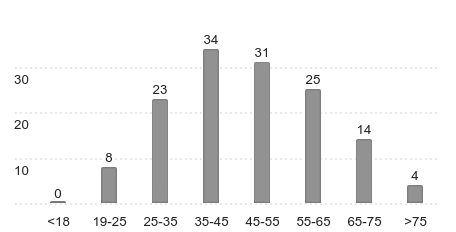


*Caption:* Estimation of awake craniotomy patients’ age according to responders (37 responders; multiple answers possible). On y axis, choice count. On x axis, age range of patients.
